# A Luminescent Proton Conductor Based on Dy_2_ SMM

**DOI:** 10.3390/molecules30051086

**Published:** 2025-02-27

**Authors:** Yingbing Lu, Yu Lei, Danpeng Cheng, Lu Long, Xiaoxuan He, Caiming Liu, Herui Wen, Suijun Liu, Shuidong Zhu

**Affiliations:** 1 College of Chemistry and Chemical Engineering, Gannan Normal University, Ganzhou 341000, China; ybluhm@163.com (Y.L.); 18586632742@163.com (Y.L.);; 2 Beijing National Laboratory for Molecular Sciences, CAS Key Laboratory of Organic Solids, Institute of Chemistry, Chinese Academy of Sciences, Beijing 100190, China; 3 School of Metallurgy and Chemical Engineering, Jiangxi University of Science and Technology, Ganzhou 341000, China

**Keywords:** luminescent, proton-conducting, single-molecule magnet

## Abstract

Multifunctional materials bearing photoluminescence, single-molecule magnet (SMM) behavior, and proton conduction have been particularly attractive for various promising applications in optics, molecular spintronics, high-density data storage, and fuel cells. However, these kinds of multifunctional systems have rarely been reported. Herein, a Dy^III^-SMM together with luminescent and proton-conducting properties, [Dy_2_(1-tza)_4_(phen)_4_]∙(ClO_4_)_2_∙(H_2_O)_2_ (**1**, 1-tza = 2-(1H-tetrazol-1-yl)acetic, phen = 1,10-phenanthroline), was prepared and structurally characterized. Complex **1** features a dinuclear structure bridged by carboxylate oxygen atoms of the 1-tza^−^ ligands, and its supramolecular network contains a 1D stacking channel. Complex **1** exhibits strong room-temperature Dy^III^ characteristic emissions and SMM behaviors. In addition, complex **1** shows a moderate proton conductivity with 4.00 × 10^−6^ S cm^−1^ at 37 °C and 100% R.H. (R.H. = Relative Humidity), which may be ascribed to the 1D-extended H-bonds in the 1D stacking channel of **1**.

## 1. Introduction

The design and construction of multifunctional coordination complexes (CCs) built by metal ions/clusters and organic ligands have attracted considerable interest because different functions, such as catalysis, sensing, optical, electrical, and magnetic properties, can exist separately or act in synergy, resulting in more efficient, flexible and smart applications [1,2,3,4]. Moreover, CCs provide a powerful platform for achieving multifunctional materials in which a variety of functions can be realized by judicious selection of metal ions and organic ligands as well as by pre-designing structures [5,6]. Within this context, the use of lanthanide ions (Ln) as the metal center is appealing as these trivalent ions possess amazing physical properties owing to their unique 4*f* electrons. On the one hand, lanthanide ions are known to present exceptional luminescent properties such as long-lived emissions, high quantum yields, and large Stokes shifts, resulting in numerous applications in white-light emission, lasers, sensing, color displays, and luminescent thermometers [7,8,9]. Moreover, Ln-based complexes show a wide range of emission from UV (e.g., Dy^3+^, Gd^3+^) and visible (e.g., Eu^3+^, Tb^3+^, Sm^3+^) to the near-infrared region (e.g., Nd^3+^, Yb^3+^, Er^3+^) [10,11,12]. In this field, the use of suitable ligands that can absorb light and transfer energy to Ln^III^ ions (so-called “antenna effect”) to enhance the luminescence of Ln^III^ ions is of importance [13]. On the other hand, the large magnetic moment and substantial magnetic anisotropy of Ln^III^ ions, especially for the Dy^III^ ion, make them very useful for the construction of high-performance single-molecule magnets (SMMs) [14,15]. Such magnets as a class of nanomagnets, which have well-defined magnetic bistability, are promising for applications in high-density data storage, quantum computing, and spintronics [16,17]. Numerous lanthanide-based polynuclear and mononuclear SMMs have been reported [18,19,20]. Previous studies showed that the magnetic anisotropy of Ln^III^ ions mainly originates from spin–orbit coupling and crystal field effects [21,22,23]. For example, the axial crystal field is necessary for a Dy-based SMM, and thus, some high-performance Dy-based complexes displaying high-energy barriers [24] or magnetic blocking temperature (*T*_B_) values near liquid nitrogen temperature have been documented [25,26]. However, these Dy organometallic complexes suffer from poor air stability. Therefore, the construction of SMMs with air stability and high performance is highly desired. Moreover, magnetic interactions in SMMs can powerfully suppress the quantum tunneling of magnetization (QTM) to enhance the effective barrier (*U*_eff_) [27]; therefore, the construction of the simple polynuclear Ln_2_ SMMs is particularly pursued because they can provide a good platform for studying magnetic exchange. Multifunctional SMMs combined with other properties are an emerging research hotspot to deeply understand magnetic behavior in SMMs and realize their applications as soon as possible [28]. A number of bifunctional SMMs, such as electrical-conducting [29,30], chiral [31], and proton-conductive−SMM [32], have been reported. Luminescent SMMs [33,34,35] are of special interest because not only the association between magnetic anisotropy and luminescence observed on the basis of the electronic structure of 4*f* metal ions [36] but also their potential uses in novel opto-magnetic devices [37,38]. Tri- or more functional SMMs have been explored to expand multifunctionality in this field [39]; however, reasonable design and preparation of more functional SMMs remains challenging.

Proton conducting materials fabricated based on CCs, which have promising applications in fuel cells, smart grids, and information processing devices inspired by biological systems [40,41,42,43], have attracted immense attention because of their unique features, such as low cost, moderate operating temperature, and an enormous structural and chemical variety [44,45]. These crystalline solids can also offer deep insight into the proton transport mechanism, which is conducive to designing and constructing high-performance proton conductors [46,47,48]. Previous studies revealed that the density of H-bonding networks in CCs is one of the key factors for proton transportation. To date, a number of CCs-based water-assisted low-temperature proton conductors with high conductivity have been prepared [49,50,51,52,53,54]; meanwhile, various CPs-based high-performance anhydrous proton conductors have been fabricated by loading guest proton carriers (e.g., imidazole, triazole, histamine) into frameworks at temperatures higher than 100 °C [55,56,57,58]. In addition, the combination of proton conduction and magnetism has attracted much attention because possible synergy may result in a new phenomenon, namely, spinprotonics [59,60]. Although many bifunctional proton-conducting magnets and luminescent magnets have been reported, trifunctional proton-conducting fluorescent magnets are extremely limited because incorporating luminescence and magnetism into porous structures with proton conduction is still challenging. Thus far, only five works on such trifunctional materials have been documented [61,62,63,64,65].

In recent years, our group has focused on investigating the luminescent, electrical, and magnetic properties of coordination complexes [66,67,68]. As part of our continuing research, in this work, the Dy^III^ ion and the 2-(1H-tetrazol-1-yl)acetic acid (1-Htza) ligand, as well as the 1,10-phenanthroline (phen) auxiliary ligand, become our first choice to construct trifunctional luminescent SMMs with proton conduction based on the following reasons: (1) the phen molecule as a N,N’bulky auxiliary ligand can block the Dy^III^ center to form low dimensionality with a nanomagnet behavior; (2) the phen ligand as an aromatic conjugate molecule can sensitize Dy^III^ emission; (3) the 1-Htza ligand possessing a tetrazole ring is an excellent proton acceptor, and has advantages to achieve a proton conductor. Herein, we report a new dinuclear trifunctional complex, namely, [Dy_2_(1-tza)_4_(phen)_4_]∙(ClO_4_)_2_∙(H_2_O)_2_ (**1**, 1-tza = 2-(1H-tetrazol-1-yl)acetic, phen = 1,10-phenanthroline), which exhibits emissive, single-molecule magnetic and proton-conductive properties.

## 2. Experimental Section

### 2.1. Materials and Instruments

2-(1H-tetrazol-1-yl)acetic acid, 1,10-phenanthroline, Dy(ClO_4_)_3_∙6H_2_O, and CH_3_CN were employed commercially without further purification. The FT-IR spectra were collected on a Nicolet Magna 750 FT-IR spectrophotometer (Thermo Fisher Scientific, Waltham, MA, USA) in the region of 4000–400 cm^−1^ using KBr pellets as bases. Elements analyses of C, H, and N were conducted on an Elementar Vario EL III microanalyzer (Elementar Analysensysteme GmbH, Langenselbold, Germany). PXRD patterns at room temperature were acquired on a Rigaku Miniflex II diffractometer (Rigaku Holdings Corporation, Tokyo, Japan) using Mo-K radiation (*λ* = 1.540598 Å). Simulated PXRD patterns were measured from the Mercury Version 1.4 software (https://www.ccdc.cam.ac.uk/solutions/software/mercury/ (accessed on 8 August 2024)). TGA measurements have been carried out on polycrystalline samples under a nitrogen atmosphere at a heating rate of 10 °C/min with the temperature ranging from 25 °C to 800 °C by Mettler Toledo thermo gravimetric analyzer (Mettler Toledo, Hongkong, China). Solid-state absorption spectra were collected on Cary 5000 UV-Vis-NIR (Agilent Technologies, Inc., Santa Clara, CA, USA) in the region of 200–800 nm. Photoluminescence analyses were conducted on an Edinburgh FL S920 fluorescence spectrometer (Edinburgh Instruments Ltd., Livingston, UK). Proton conductivity measurements were conducted via a quasi-four-electrode AC impedance technique equipped with a Solartron 1260 impedance/gain-phase analyzer (AMETEK Scientific Instruments, Berwyn, PA, USA). The ground microcrystalline samples were compressed to 0.78 mm in thickness and 2.5 mm in diameter with a pressure of 0.1 GPa. Two sides of the pellet were attached to a couple of gold wires with gold paste. The sample pellets were recorded as the temperatures increased from 25 to 37 °C and/or as the relative humidities (RH) rose from 60% to 100% over a frequency range of 1–107 Hz. The proton conductivity of the samples was deduced from the Debye semicircle by the equation σ = *L*/(*RS*), where *L* (cm) is the thickness, *R* (Ω) is the resistance, and *S* (cm^2^) is the cross-sectional area. Magnetic susceptibilities were collected by means of a Quantum Design PPMS model 6000 magnetometer (Quantum Design, San Diego State, CA, USA), and the diamagnetic contributions of the empty container have been deducted. The experimental susceptibilities were corrected for diamagnetism by using Pascal’s constants.

### 2.2. Synthesis of [Dy_2_(1-tza)_4_(phen)_4_]∙(ClO_4_)_2_∙(H_2_O)_2_ *(**1**)*

Compound **1** was synthesized by a solvothermal approach. A mixture of Dy(ClO_4_)_3_∙6H_2_O (0.2 mmol, 0.092 g), 2-(1H-tetrazol-1-yl)acetic acid (0.40 mmol, 0.052 g), 1,10-phenanthroline (0.40 mmol, 0.072 g), and CH_3_CN (10 mL) was sealed into a 25 mL Teflon-lined stainless container under autogenous pressure. The reaction mixture was kept at 180 °C for 3 days and then cooled to room temperature at a rate of 3 °C/h. Colorless column crystals of **1** appropriate for X-ray analyses were prepared. Yield: 38% (based on Dy) for **1**. Anal. Calcd (C_60_H_48_Cl_2_Dy_2_N_24_O_18_, %): C, 40.22; H, 2.70; N, 18.77; Found (%): C, 41.08; H, 2.79; N, 18.42. FT-IR peaks (KBr, cm^−1^) for **1**: 3855 w, 3636 w, 3136 w, 1670 vs, 1517 m, 1438 s, 1395 s, 1323 m, 1255 w, 1105 vs, 973 w, 848 s, 803 m, 727 s, 686 m, 627 m, 581 w, 439 w.

### 2.3. X-Ray Single-Crystal Structure Determination

A single crystal of complex **1**, mounted on a Bruker SMART APEX CCD diffractometer (Bruker, Billerica, MA, USA), was collected by adopting graphite-monochromated Mo-K radiation (*λ* = 0.71073 Å) at 293 K. The diffraction datasets were collected and reduced by CrysAlisPro (version 1.171.38.46) [69]. The structures were solved by a direct method by using SHELX-2014 within Olex2 [70] and refined on *F^2^* with full-matrix least-squares techniques [71]. Hydrogen atoms were added geometrically and refined using a riding model. All non-H atoms were located by different Fourier maps and subjected to anisotropic refinement. No higher space groups for **1** were available, adopting the Platon software 81024 from the IUcr website (http://www.iucr.org/ (accessed on 16 October 2024)). The crystallographic data, selected bond distances, and bond angles are summarized in Tables S1 and S2 in ESI, respectively.

## 3. Results and Discussion

### 3.1. Structural Description and Discussion

**Figure 1:**
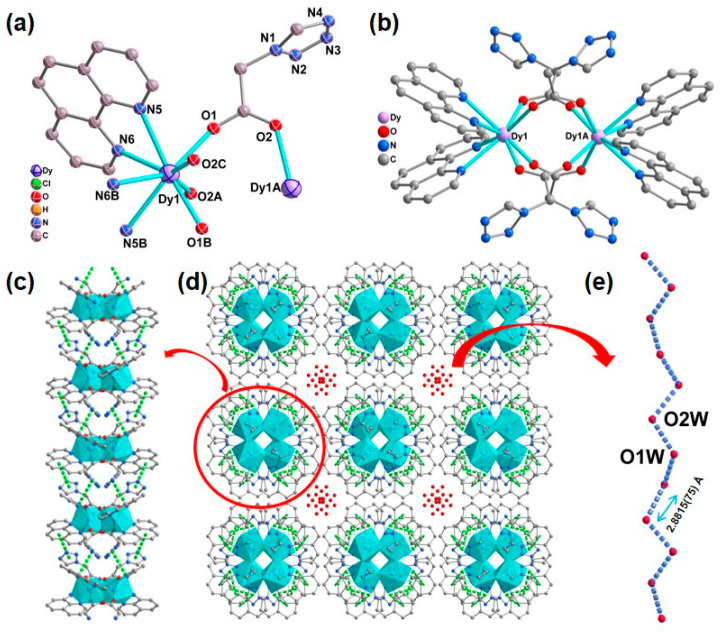
(**a**) ¼ part of the binuclear molecule of **1** with 40% thermal ellipsoids. Symmetry codes: A: − *x*, − *y +* 1/2, + *z*; B: − *x* + 1/4, − *y* + 1/4, − *z* + 1/4; C: *x* +1/4, *y* − 1/4, − *z* + 1/4. The H atoms, perchlorate anion, and lattice water molecules are omitted for clarity; (**b**) Drawing of the dinuclear structure of **1**; (**c**) One-dimensional supramolecular chains constructed by *π*⋯*π* interactions (green dashed lines) in **1** along *a* direction; (**d**) The stacking supramolecular framework constructed via *π*⋯*π* interactions (green dashed lines) in **1** in the *ab* plane, perchlorate anion are omitted for clarity; (**e**) The 1D extended hydrogen bonding chain in **1**.

X-ray crystallographic analysis revealed that complex **1** crystallizes in the *I4_1_/acd* space group with a dinuclear structure; ¼ part of the binuclear molecule of **1** comprises half of Dy^III^ ions, one phen molecule, one 1-tza^−^ ligand, half of the disordered perchlorate anion, and half of disordered lattice water molecule. The Dy^III^ center of **1** is eight-coordinated via four carboxylate oxygen atoms from four different 1-tza^−^ ligands (O1, O1B, O2A, O2C) and four nitrogen atoms from two different chelated phen molecules (N5, N5B, N6, N6B). Using the *SHAPE* 2.1 program [72], the coordination geometry of the Dy^III^ center can be described as a triangular dodecahedron coordinated geometry with a continuous shape measurement (*CShM*) value of 0.353 (Figure 1 and Figure S1 and Table S3 in ESI). In **1**, the Dy−O/N bond lengths are within 2.2880(19)−2.562(2) Å, which are similar to those Dy−O/N based compounds [73,74]. Two neighboring Dy^III^ cations are bridged via four carboxyl groups from four *μ*_2_-*η*^1^:*η*^1^-based 1-tza^−^ ligands, leading to a lanthanide dimer (Figure 1b). These adjacent dimers are connected via face-to-face *π*-*π* stacking interactions through phenyl rings of phen ligands and tetrazole rings of 1-tza^−^ ligands, forming a 1D supramolecular chain with the centroid-to-centroid distances of 3.841(3) and 3.708(3) Å, respectively (Figure 1c). The lattice water molecules fill voids in the supramolecular framework of **1** (Figure 1d). Moreover, as shown in Figure 1d,e, a 1D extended hydrogen bonding chain via water molecules (O1W⋯O2W = 2.8815(75) Å) is formed within the stacking pores of supramolecular network **1**. In **1**, the ClO_4_^−^ anions fill in the accessible voids of these 1D supramolecular chains along the ac plane for charge balance (Figure S2 in the ESI).

### 3.2. Thermal Stability and UV Spectra

**Figure 2:**
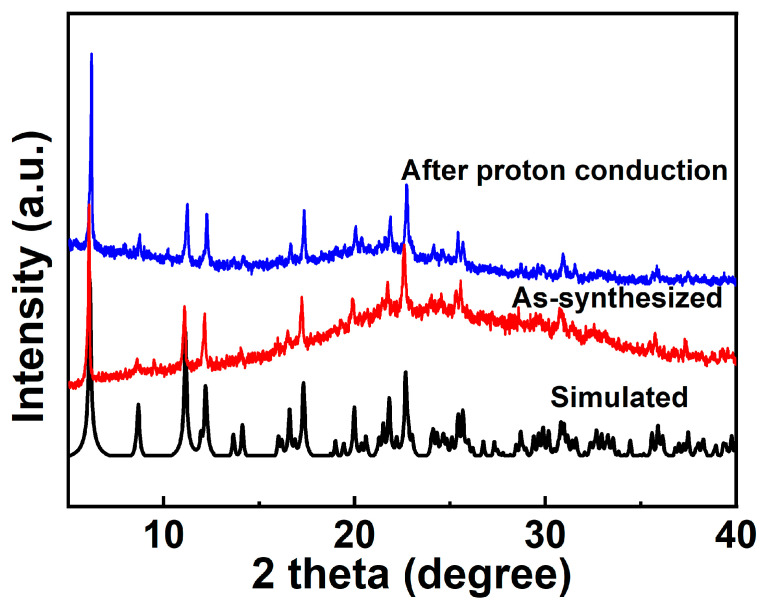
PXRD of compound **1** after proton conduction.

The measured PXRD patterns of the as-synthesized **1** well match the simulated patterns generated from the results of single-crystal diffraction data, indicating the purity of the product (Figure 2). Thermogravimetric analysis (TGA) was carried out to characterize the thermal stability of complex **1**. As shown in Figure S3, the TGA curve shows that the weight is reduced by 1.98% within 25–78 °C, which could be attributed to the loss of two lattice waters (calcd. 2.01%). With further increase in the temperature, the framework gradually decomposes, and the weight decreases by 40.85% (calcd. 40.32%) at 78–395 °C, which is assigned to the removal of four 1,10-phenanthroline molecules in **1**. Pyrolysis occurs above 480 °C due to the collapse of the network. In the DTA diagram of 1, there are three exothermic peaks near 50 °C, 299 °C, and 341 °C, which correspond to the release of two lattice water molecules and the loss of four phen molecules, respectively. As shown in Figure S4, the absorption spectra of complex **1** was consistent with that of 1,10-phenanthroline (phen).

### 3.3. Magnetic Properties

**Figure 3:**
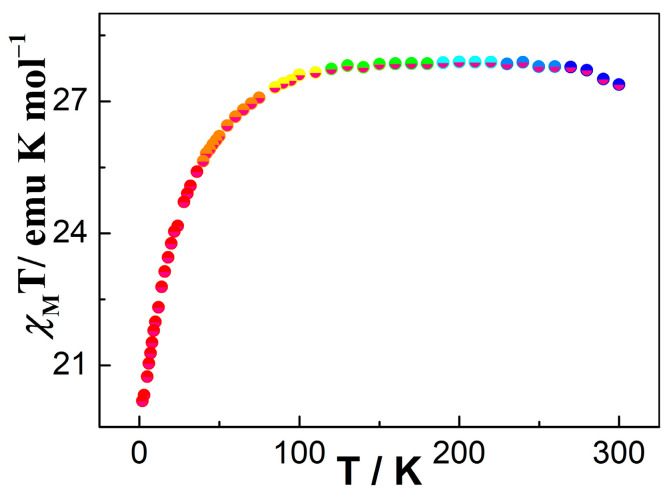
Temperature dependence of the magnetic susceptibility for complex **1** between 2 and 300 K.

Variable-temperature dc magnetic susceptibility measurements were performed for **1** in the temperature range of 2 to 300 K under an applied magnetic field of 1000 Oe (Figure 3). The χ_M_T value at 280 K for **1** is 27.32 cm^3^ K mol^−1^, which is close to two isolated Dy^III^ ions at room temperature (^6^H_15/2_, S = 5/2, L = 5, J = 15/2, g = 4/3, C = 28.34 cm^3^ K mol^−1^) [75]. In the 300–100 K range, the χ_M_T product reduces smoothly; at below 100 K, the χ_M_T for **1** further decreases to the lowest value of 20.19 cm^3^ K mol^−1^ at 2 K. This behavior can be ascribed to possible weak inter-complex antiferromagnetic coupling and/or the thermal depopulation of Stark sublevels of Dy^III^ ion [76]. For complex **1**, the magnetization value was investigated within 2–6 K (Figure S5b). The plot of M versus H displays a steady increase with increasing field to reach the values of 10.56 Nβ for **1** at 6 K, which is lower than the theoretical value of 20 Nβ for two Dy^III^ ions (Figure S5a) [77]. Moreover, as shown in Figure S5b, the curve of M vs. H/T displayed a non-superimposable nature, suggesting that significant magnetic anisotropies exist in **1** [78].

**Figure 4:**
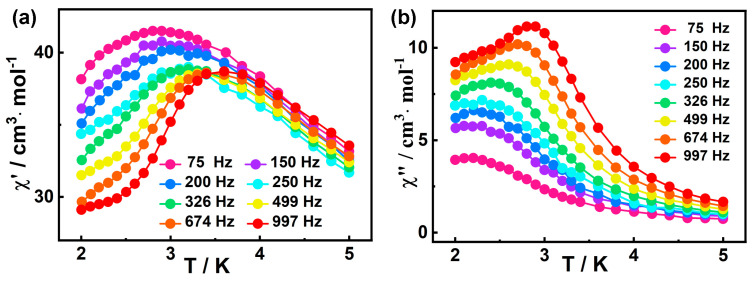
Frequency-dependent *χ*′ (**a**) and *χ*″ (**b**) signals for **1** at 2000 Oe dc field.

The frequency dependence of ac susceptibilities were also measured to investigate their dynamic magnetic behavior. Based on the field scan results (Figure S6), the optimal field for studying the slow magnetic relaxation of **1** appears to be 1400 Oe. However, the out-of-phase (*χ*″) peak of ac magnetic susceptibility was not observed at 3 K and *H*_dc_ = 1400 Oe (Figure S7), indicating 1400 Oe is not the optimal external magnetic field. Therefore, we tried to choose a common 2000 Oe external field to eliminate QTM. Fortunately, as shown in Figure 4a,b, the out-of-phase (*χ*″) peaks at all frequencies (10 Hz−1399 Hz) appear under this external magnetic field, so the external magnetic field is appropriate. The in-phase (*χ*′) and out-of-phase (*χ*″) signals of **1** show frequency dependencies within 2.0–5.0 K, indicating the SMM behavior of **1**. [79].

**Figure 5:**
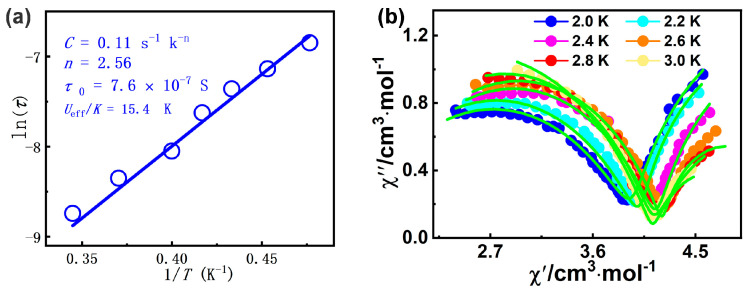
(**a**) The ln *τ* vs. 1/*T* graph for **1**; (**b**) Cole-Cole plot for **1** under 2000 Oe dc field (solid lines: fitting results).

The ln(*τ*)-1/*T* curve could be fitted with the equation including Raman and Orbach processes, *τ*^−1^ = *CT*^n^ + *τ*_0_^−1^ exp(−*U*_eff_/*kT*) [80], giving *n* = 2.56, *C* = 0.11 s^−1^ K^−2^.^56^, *U*_eff_/*k* = 15.4 K, and *τ*_0_ = 7.6 × 10^−7^ s for **1** (Figure 5a). The energy barrier of **1** can be comparable to that of the complex [Dy_4_(*μ*_4_-O)-(HL^3^)_4_(H_2_L^3^)_2_]∙3H_2_O∙EtOH∙CH_3_CN (H_3_L^3^ = 3-(((2-hydroxynaphthaen-1-yl)methylene)amino)-propane-1,2-diol, *U*_eff_ = 2.6 K) [81]. The obtained *τ*_0_ value falls in the characteristic range for SMMs (10^−5^ to 10^−11^ s) [61]. Furthermore, the Cole–Cole diagram (*χ*′_M_ vs. *χ*′′_M_) of complex **1** between 2.0 and 3.0 K exhibits dual magnetic relaxation characteristics (Figure 5b), which the left and the right semi-circular curves correspond to the fast relaxation (FR) phase and the slow relaxation (SR) phase, respectively. By using CC–FIT2 software (https://www.nfchilton.com/ (accessed on 5 October 2024)), Table S4 summarizes the least-squares fitting parameters. The *α*_1_ and *α*_2_ values are not small, and the *α*_1_ value (0.203–0.326) is slightly larger than the *α*_2_ values (0.000–0.274); the results show that the SR phase has a relatively narrow distribution of relaxation time with respect to the FR phase.

### 3.4. Proton Conduction

**Figure 6:**
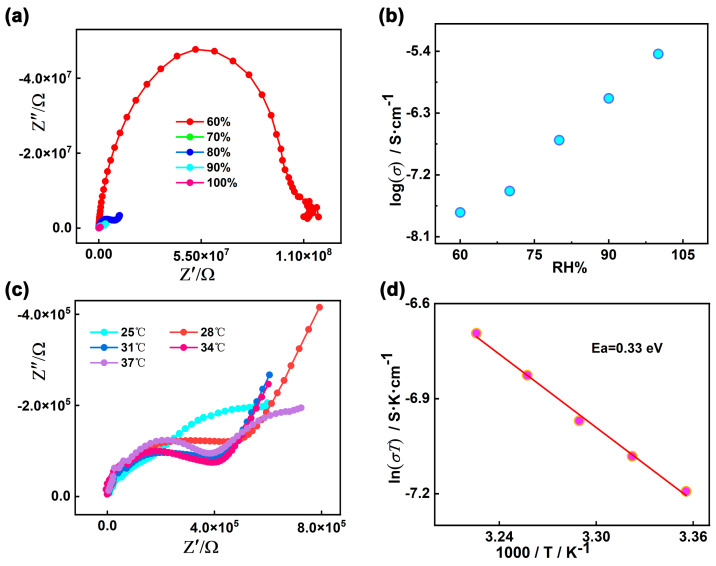
(**a**) Nyquist plot for **1** at 35 °C under different RH levels; (**b**) Plot of proton conductivity for **1** vs. RH at 35 °C; (**c**) Nyquist plot for **1** at different temperatures under 100% RH; (**d**) Plots of ln(*σT*) vs. 1000/*T* for **1** under 100% RH.

The proton conductivities of **1** were carried out using Alternating-current impedance measurement at different RH levels (60−100%) and varied temperatures (25–37 °C). The semicircles in the high-frequency region and inclined tails in the low-frequency range in the Nyquist plots are displayed in Figure 6a,c, indicating that **1** is a typical proton conductive behavior [82,83,84]. At the constant temperature of 35 °C, the diameters of the semicircles notably decreased with increasing in RH from 60% to 100%, suggesting the proton conductivity values (*σ*) are closely dependent on humidity (Figure 6b and Figure S8). As demonstrated in Figure S9 and Table S5, the conductivity (*σ*) of complex **1** increases by two orders of magnitude with the σ from 1.79 × 10^−8^ S cm^−1^ at 60% RH to 3.63 × 10^−6^ S cm^−1^ at 100% RH under 35 °C. The behavior is similar with other many water-mediated proton-conductive CPs [85,86]. The temperature-dependent proton conductivitywas investigated under different temperatures and fixed humidity. Under 100% RH, the conductivity (*σ*) of complex **1** slowly increased from 2.52 × 10^−6^ S cm^−1^ at 25 °C to reach a maximum value of 4.00 × 10^−6^ S cm^−1^ at 37 °C (Figure 6c, Figures S10 and S11, and Table S6). The optimal proton conductivity for complex **1** is comparable to those of previously reported CPs-based proton conductor, for instance, UiO-66-NH_2_ (3.5 × 10^−6^ S cm^−1^ at 80 °C and 98% RH) [87], [Zn_3_(ssa)_2_(1,4-bib)_3_∙4H_2_O]_n_ (H_3_ssa = 5-sulfosalicylic acid; 1,4-bib =1,4-bis (1H-imidazol-1-yl)benzene], 6.26 × 10^−6^ S cm^−1^, At 60 °C and 95% RH) [88], 5,10,15, 20-tetrakis[p-phenylphosphonic acid] (GTUB5, 3.00 × 10^−6^ S cm^−1^ at 75 °C and 75% RH) [89]. To further explore the proton migration mechanism of **1**, *E*_a_ was calculated by fitting to the Arrhenius equation: *σT* = *A* exp(−*E*_a_/*k*_B_*T*), where *E*_a_, *σ*_0_, *k*_B_ and *T* are the activation energy for conduction, pre-exponential factor, Boltzmann’s constant and the absolute temperature, respectively. The previous reports showed thatthe Grotthuss mechanism (usually *E*_a_ < 0.4 eV) involves the proton transport along the hydrogen bonding network [90,91] and the vehicle mechanism (usually *E*_a_ > 0.5 eV) is governed by the migration of protons through the assistance of a moveable species (e.g., NH_3_, H_2_O) [92,93,94]. The *E*_a_ value of **1** is 0.33 eV, indicating that the proton transfer of **1** is mediated via the Grotthuss mechanism (Figure 6d). The proton conduction is related to the extended 1D H-bonds formed by the lattice water molecules within the pores of supramolecular **1**. Furthermore, the structural stability of **1** was confirmed by the PXRD spectra before and after the conductivity measurements, and there are no obvious changes and no fade on the crystal surface (Figure 2 and Figure S12).

### 3.5. Photoluminescent Properties

**Figure 7:**
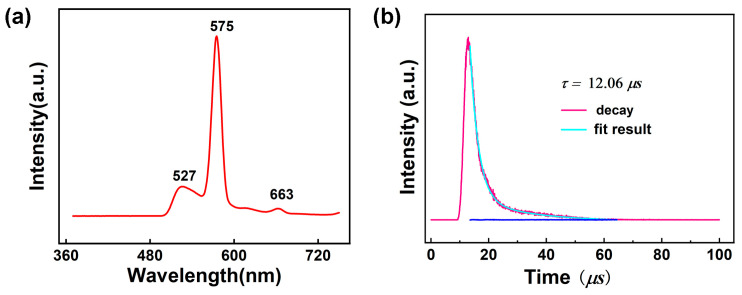
(**a**) Solid-state emission spectra for **1** at room temperature; (**b**) Decay curves of **1**.

Lanthanide complexes display intriguing photoluminescent properties originating from unique 4*f* electrons of these trivalent cations [95,96]. Consequently, luminescent Ln-based complexes have gained extensive attention because of their wide applications in sensors, biolabeling, solid-state lasers, light-emitting diodes, optical switches, and so on [97,98]. The free 1-Htza ligand shows photoluminescent emission bands at 423 nm upon photoexcitation at 278 nm, which can be assigned to the ligand-centered π-π* transitions (Figure S13). The solid-state luminescence spectrum of **1** was tested at room temperature to explore the luminescence behavior of **1**. As illustrated in Figure 7a and Figure S14, upon the excitation at 350 nm, **1** displays an emission spectrum containing three peaks at 527 nm, 573, and 662 nm. The round peak of 527 nm may be ascribed to the emission of the 1-tza^–^ ligand because the free 1-tza^–^ ligand shows emission bands at 525 nm upon photoexcitation at 350 nm (Figure S15). The peaks of 575 and 663 nm are attributed to ^4^*F*_9/2_→^6^*H*_13/2_ and ^4^*F*_9/2_→^6^*H*_11/2_ transitions of the Dy^III^ ion, respectively. According to the literature [99], the singlet energy level (^1^ππ*) and the lowest triplet energy level of phen ligand (^3^ππ*) are 31,000 cm^−1^ and 22,100 cm^−1^, respectively. The energy difference (∆*E* = ^3^ππ*–^5^*D*_4_) between the lowest triplet level of phen and the resonant energy level of the Dy^III^ ions was calculated to be 1397 cm^−1^, indicating that the value matches well with the 4*f* levels Dy^III^ ions. Thus, the 1,10-phen can sensitize Dy^III^ emission effectively by “antenna effect”. The luminescence lifetime of **1** was analyzed using the strongest emission (573 nm) and excitation (350 nm) within the visible region by fitting with the double-exponential function: *I* = *A* + *B*_1_exp(−*t*/*τ*_1_) + *B*_2_exp(−*t*/*τ*_2_) (*τ*_1_ and *τ*_2_ represent the fast and slow components of the luminescent lifetimes, and *A*, *B*1, and *B*2 are the fitting parameters) [100] (Figure 7b), and the average lifetime value is around 12.06 *μ*s for **1** based on *τ* = (*B*1*τ*_1_^2^ + *B*2*τ*_2_^2^)/(*B*1*τ*_1_ + *B*2*τ*_2_) (*τ*_1_ = 2915.22 ns, *τ*_2_ = 20,721.68 ns, *B*1 = 1702.45 and *B*2 = 252.79) [101], which can be comparable to those reported for Dy^III^ complexes [102]. In addition, the luminescence quantum yield of **1** was measured but is extremely low and almost negligible, which is consistent with the reported Dy-based complex {[Ln(*μ*_4_-pmdc)(NO_3_)(H_2_O)]∙H_2_O}_n_ (pmdc = pyrimidine-4,6-dicarboxylic acid) [103].

## 4. Conclusions

In summary, a new multifunctional Dy^III^ complex with a dinuclear structure was successfully obtained by using simple ligands with 2-(1H-tetrazol-1-yl)acetate ligand and 1,10-phenanthroline auxiliary ligand. The molecular materials exhibit trifunctionalities with Dy^III^ characteristic emissions and a field-induced SMM behavior, together with a moderate proton conductivity related to the 1D extended H-bonds constructed by the lattice water molecules. This work provides new insights into the design of multifunctional materials that simultaneously exhibit photoluminescence, SMM, and proton conduction.

## Data Availability

X-ray crystallographic data file in CIF format, PXRD, IR spectra, selected bond distances, and angles for **1**. The TGA plot of **1**. Solid-state fluorescence excited spectra of **1**. Magnetic data of **1**. The proton conductivity of **1** under different RHs and temperatures. Solid-state fluorescence emission spectra of the free 1-Htza ligand. CCDC number: 2190704 for **1**.

## Supplementary Materials

The following supporting information can be downloaded at: https://www.mdpi.com/article/10.3390/molecules30051086/s1. Table S1. Crystal data for **1.** Table S2. Selected Bond Lengths (Å) and Bond Angles (^o^) for **1.** Table S3. Summary of SHAPE analysis of Dy1 in **1.** Figure S1. The triangular dodecahedron (TDD-8) coordination geometry of Dy^III^ in **1**. Figure S2. The 3D network of **1** in which the perchloric acid filled in one channel along *c* axes (H at oms are omitted for clarity). Figure S3. TGA of **1** from 25 °C to 800 °C. Figure S4. Solid-state absorption spectra of complex **1**, and phen and 1-Htza ligands. Figure S5. (a) *M* versus *H* plot of **1** at 2 K; (b) Experimental *M* versus *H*/*T* plots of **1** Figure S6 The in-phase (*χ*′) and out-of-phase (*χ*″) components of ac magnetic susceptibility under variable dc fields for **1** at 3 K and frequency with 1399 Hz. Figure S7. ac susceptibility measurements at frequency with 1399 Hz for **1** at 3 K and *H_dc_* = 1400 Oe. Table S4. Linear combination of two modified debye model fitting parameters from 3 to 7 K at *H*_dc_ = 2000 Oe. Figure S8. Nyquist plot for **1** Aat 35 °C under (a) 60%, (b) 70%, (c) 80%, (d) 90%, (e) 100% RH. Figure S9. The best-fit result of Nyquist plot for **1** at 35 °C under different RH levels. Table S5. The proton conductivity of **1** at 35 °C under variable relative humidity (RH). Figure S10. Nyquist plot for **1** at (a)25 °C, (b) 28 °C, (c) 31 °C, (d) 34 °C and (e) 37 °C under 100% RH. Figure S11. The best-fit result of Nyquist plot for **1** at different temperatures under 100% RH. Table S6. The proton conductivity of **1** at 100% RH under variable temperature. Figure S12. The photograph of crystals of complex **1** after exposed to 25−37 °C and 60−100% RH conditions during the whole proton conductivity measurements. Figure S13. Solid-state excited (a) and emission (b) spectra free Htza ligand. Figure S14. Solid-state excitation spectra for **1** at room temperature. Figure S15. Soild-state emission spectra of the free 1-Htza ligand at room-temperature. Figure S16. IR spectra for **1.**
