# Supplementary material for: A Luminescent Proton Conductor Based on Dy2 SMM
Source: Molecules. 2025 Feb 27;30(5):1086. doi: 10.3390/molecules30051086 (PMC11901984; doi:10.3390/molecules30051086)
Supplement: Supplementary file 1 [file molecules-30-01086-s001.zip › molecules-3324435-supplementary.pdf]

Supporting Information

A Luminescent Proton Conducor Based on Dy_2 SMM

Yingbing Lu [1,*], Yu Lei[1], Danpeng Cheng[1], Lu Long[1], Xiaoxuan He[1], Caiming Liu[2,*], Herui Wen[3], Suijun Liu[3], and Shuidong Zhu[1,*]

[1] College of Chemistry and Chemical Engineering, Gannan Normal University, Ganzhou 341000, China

[2] Beijing National Laboratory for Molecular Sciences, CAS Key Laboratory of Organic Solids,

Institute of Chemistry, Chinese Academy of Sciences, Beijing 100190, China.

[3] School of Metallurgy and Chemical Engineering, Jiangxi University of Science and Technology,

Ganzhou 341000, China.

[*] E-mail: ybluhm@163.com(Y.-B.L.); cmliu@iccas.ac.cn(C.-M.L.); zsd2002@sina.com(S.-D.Z.)

Table S1. Crystal data for **1.**

Table S2. Selected Bond Lengths (Å) and Bond Angles (°) for **1.**

Table S3. Summary of SHAPE analysis of Dy1 in **1.**

Fig. S1. The triangular dodecahedron (TDD-8) coordination geometry of Dy^{III} in **1**.

Fig. S2. The 3D network of **1** in which the perchloric acid filled in one channel along *c* axes (H at oms are omitted for clarity).

Fig. S4. Solid-state absorption spectra of complex **1**, and phen and 1-Htza ligands.

Fig. S5. (a) *M* versus *H* plot of **1** at 2 K; (b) Experimental *M* versus *H*/*T* plots of **1**

Fig. S6 The in-phase (χ') and out-of-phase (χ'') components of ac magnetic susceptibility under variable dc fields for **1** at 3 K and frequency with 1399 Hz.

Fig. S7. ac susceptibility measurements at frequency with 1399 Hz for **1** at 3 K and H_{dc} = 1400 Oe.

Table S4. Linear combination of two modified debye model fitting parameters from 3 to 7 K at H_{dc} = 2000 Oe.

Fig. S8. Nyquist plot for **1** at 35 °C under (a) 60%, (b) 70%, (c) 80%, (d) 90%, (e) 100% RH.

Fig. S9. The best-fit result of Nyquist plot for **1** at 35 °C under different RH levels.

Table S5. The proton conductivity of **1** at 35 °C under variable relative humidity (RH).

Fig. S10. Nyquist plot for **1** at (a)25 °C, (b) 28 °C, (c) 31 °C, (d) 34 °C and (e) 37 °C under 100% RH.

Fig. S11. The best-fit result of Nyquist plot for **1** at different temperatures under 100% RH.

Table S6. The proton conductivity of **1** at 100% RH under variable temperature.

Fig. S12. The photograph of crystals of complex **1** after exposed to 25−37 °C and 60−100% RH conditions during the whole proton conductivity measurements.

Fig. S13. Solid-state excited (a) and emission (b) spectra free Htza ligand.

Fig. S14. Solid-state excitation spectra for **1** at room temperature.

Fig. S15. Soild-state emission spectra of the free 1-Htza ligand at room-temperature.

Fig. S16. IR spectra for **1.**

Table S1. X-ray Diffraction Crystallographic Data for **1.**

Complex **1**			
Formula	$C_{60}H_{48}Cl_2Dy_2N_{24}O_{18}$	μ (mm^{-1})	2.226
Fw	1789.12	F (000)	7088.0
Temp (K)	293	Reflns collected	101463
Crystal system	tetragonal	Independent reflns	4126
Space group	*I*4$_1$/*acd*	R_{int}	0.0507
a, Å	28.8509(9)	Theta range, °	5.504–55.034
b, Å	28.8509(9)	Params/restraints/data	336/263/4126
c, Å	17.2431(6)	R_1 [$I > 2\sigma$(I)]	0.0258
D_c, g/cm^3	1.656	wR_2 (all data)	0.0559
V, Å^3	14352.7(10)	GOF on F^2	1.086
Z	8	ρ_{max}/ρ_{min}, *e* Å^{-3}	1.02/–0.45

$^aR1 = ||F_o|–|F_c||/|F_o|$; $^bwR2 = [w(F_o^2–F_c^2)^2]/[w(F_o^2)^2]^{1/2}$

Table S2. Selected Bond Lengths (Å) and Bond Angles (°) for **1.**

Bond	Lengths (Å)	Bond	Lengths (Å)
Dy(1)-O(1)	2.2879(19)	Dy(1)-N(6)	2.562(2)
Dy(1)-O(1)#1	2.2880(19)	Dy(1)-N(6)#1	2.562(2)
Dy(1)-O(2)#3	2.3333(19)	Dy(1)-N(5)	2.562(2)
Dy(1)-O(2)#2	2.3333(19)	Dy(1)-N(5)#1	2.562(2)
Angles	Angles (°)	Angles	Angles (°)
O(1)-Dy(1)-O(1)#1	105.82(10)	O(1)-Dy(1)-N(6)	98.20(8)
O(1)-Dy(1)-O(2)#2	77.07(7)	O(1)#1-Dy(1)-N(6)	140.35(7)
O(1)#1-Dy(1)-O(2)#2	78.50(7)	O(1)#1-Dy(1)-N(6)#1	98.20(8)
O(1)#1-Dy(1)-O(2)#3	77.07(7)	O(1)#1-Dy(1)-N(5)	153.61(8)
O(1)#1-Dy(1)-O(2)#3	77.07(7)	O(1)#1-Dy(1)-N(5)#1	73.84(7)
O(1)-Dy(1)-N(6)#1	140.35(7)	O(1)-Dy(1)-N(5)#1	153.62(8)

Symmetry Codes for **1**, #1 − *x* + 1/4, − *y* + 1/4 , − *z* + 1/4; #2 − *x*, − *y* + 1/2, +*z*; #3 *x* + 1/4, *y* −1/4 , − *z* + 1/4；

Table S3. Summary of *SHAPE* analysis of Dy1 for **1.**

label	shape	symmetry	Distortion(τ)
OP-8	Octagon	D8h	32.242
HPY-8	Heptagonal pyramid	C7v	23.703
HBPY-8	Hexagonal bipyramid	D6h	15.162
CU-8	Cube	Oh	7.947
SAPR-8	Square antiprism	D4d	2.495
**TDD-8**	**Triangular dodecahedron**	**D2d**	**0.353**
JGBF-8	Johnson gyrobifastigium J26	D2d	15.516
JETBPY-8	Johnson elongated triangular bipyramid J14	D3h	29.740

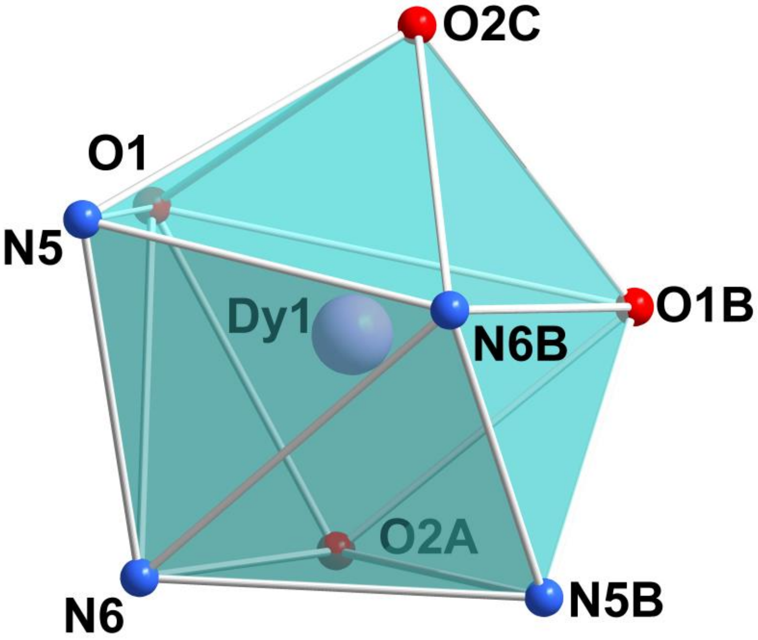

Fig. S1. The triangular dodecahedron (TDD-8) coordination geometry of Dy^{III} in **1**.

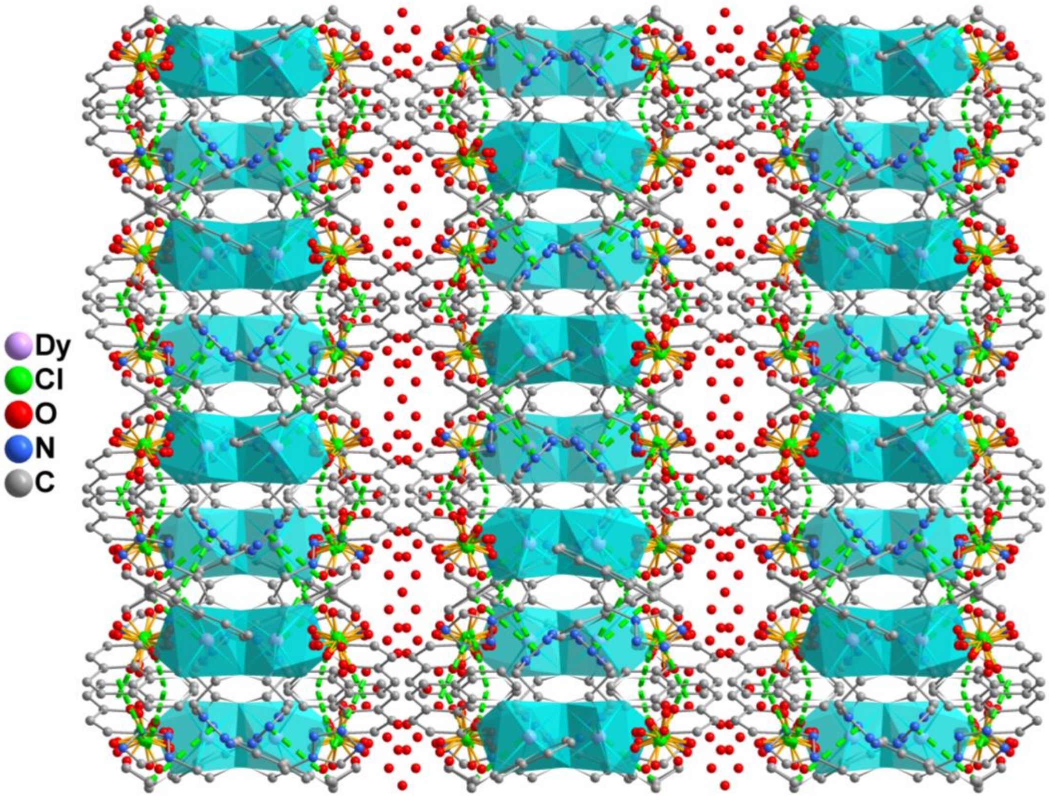

Fig. S2. The stacking supramolecular framework of **1** *via* $\pi\cdots\pi$ interactions in which the perchloric anions filled in one channel along *b* axes (H at oms are omitted for clarity)

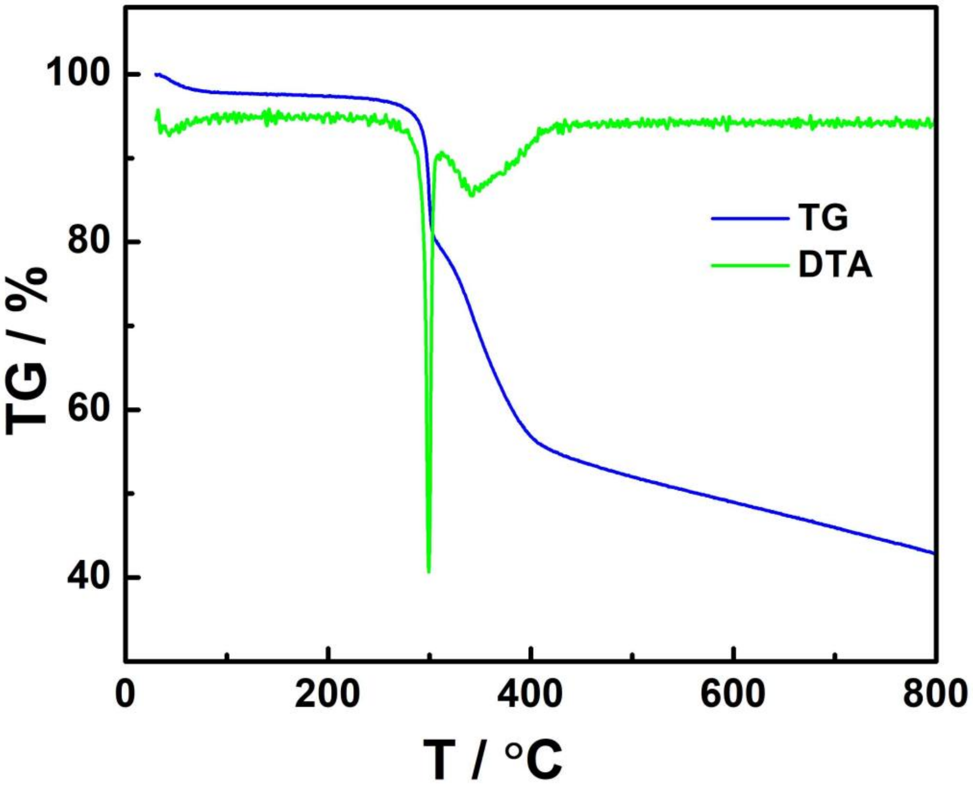

Fig. S3. TGA and DTA plots of **1**

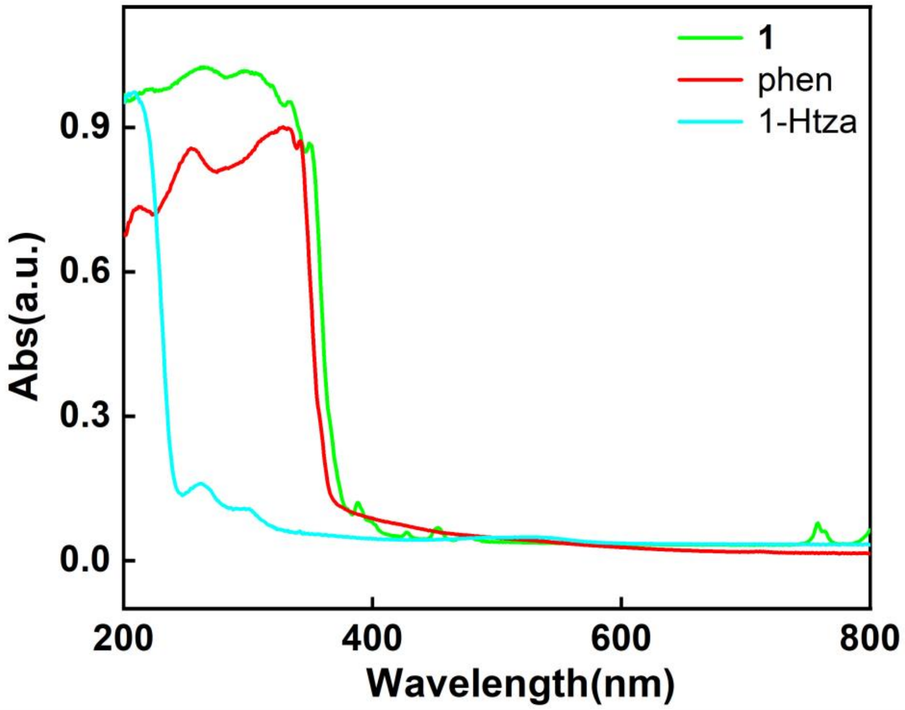

Fig. S4. Solid-state absorption spectra of complex **1**, and phen and 1-Htza ligands

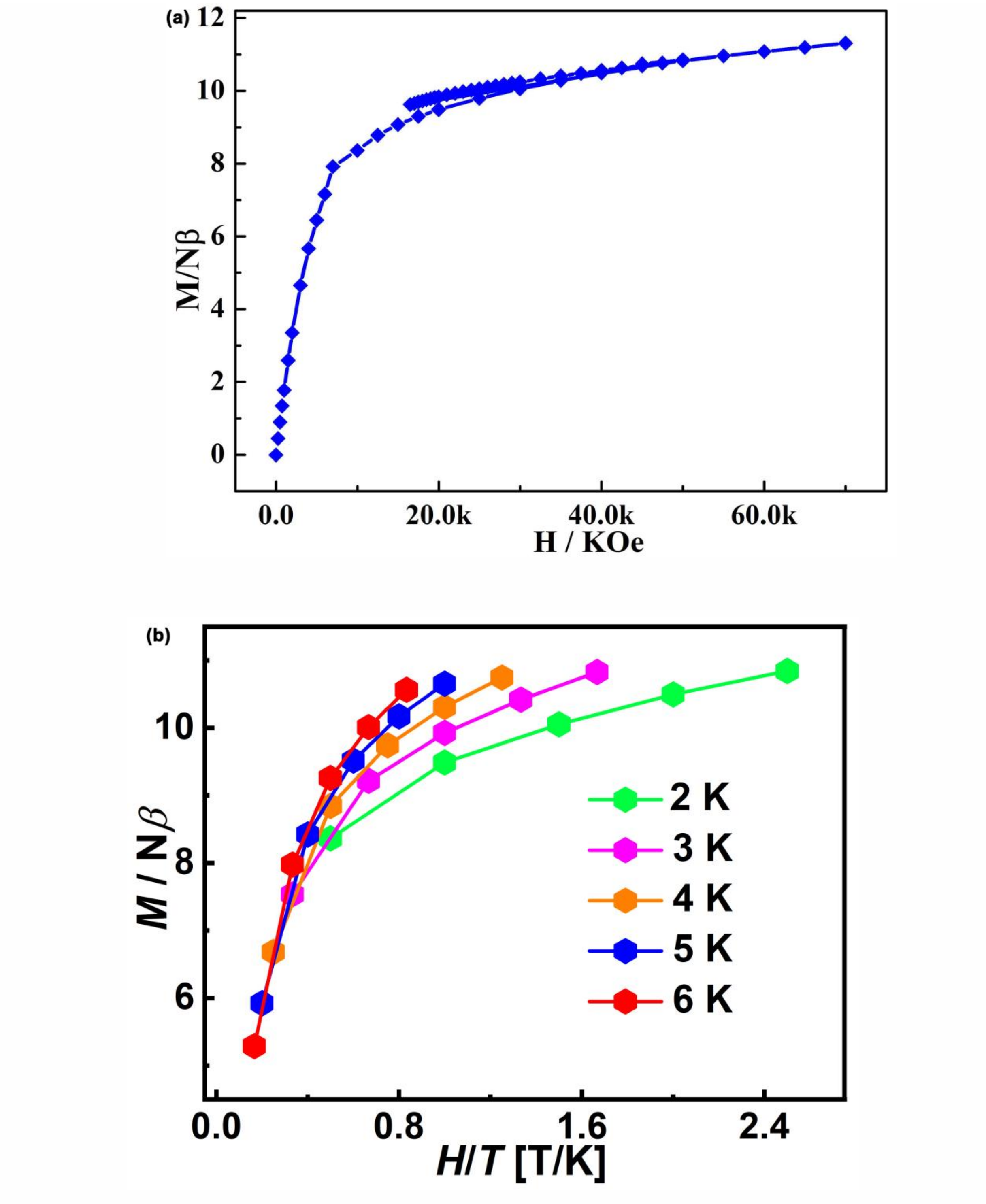

Fig. S5. (a) *M* versus *H* plot of **1** at 2 K; (b) Experimental *M* versus *H*/*T* plots of **1**

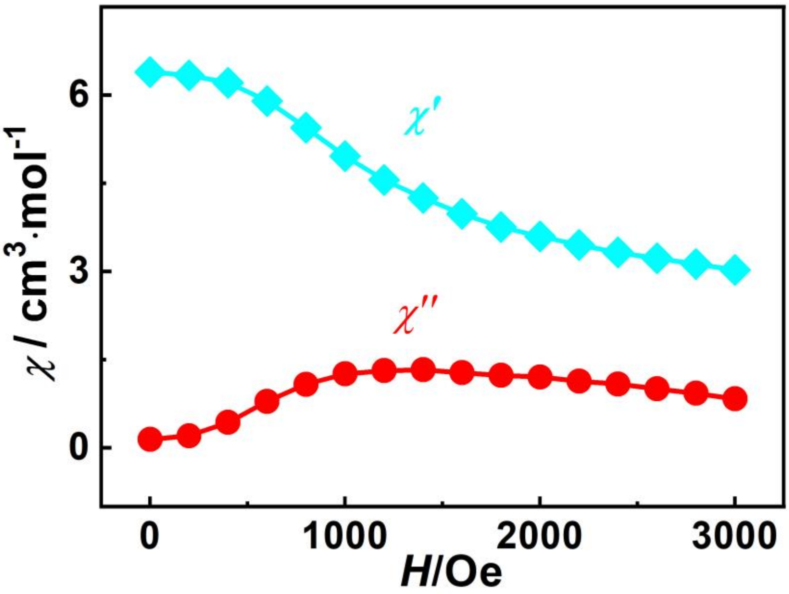

Fig. S6. The in-phase (χ') and out-of-phase (χ'') components of ac magnetic susceptibility under variable dc fields for **1** at 3 K and frequency with 1399 Hz.

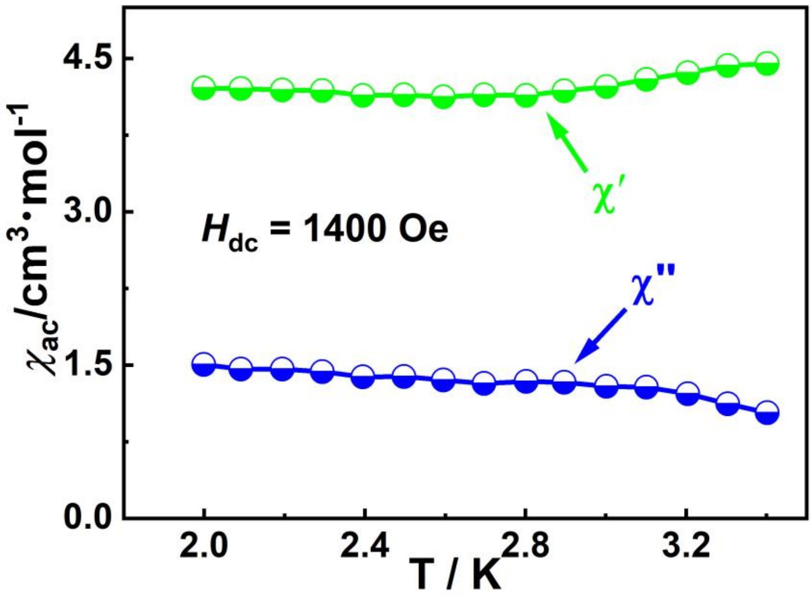

Fig. S7. ac susceptibility measurements at frequency with 1399 Hz for **1** at 3 K and H_{dc} = 1400 Oe.

Table S4. Linear combination of two modified debye model fitting parameters from 3 to 7 K at H_{dc} = 2000 Oe.

T/K	τ_1 / s	α_1	τ_2 / s	α_2
2.0	0.00022(3)	0.32642(2)	0.35812(4)	0.09237(3)
2.2	0.00019(3)	0.29833(3)	0.32413(5)	0.04990(4)
2.4	0.00020(4)	0.24362(3)	0.45003(7)	0.11742(6)
2.6	0.00018(4)	0.22072(6)	0.16357(8)	0.00000(7)
2.8	0.00013(5)	0.20308(3)	0.48255(4)	0.27430(8)
3.0	0.00007(3)	0.24863(5)	0.25858(3)	0.15352(4)

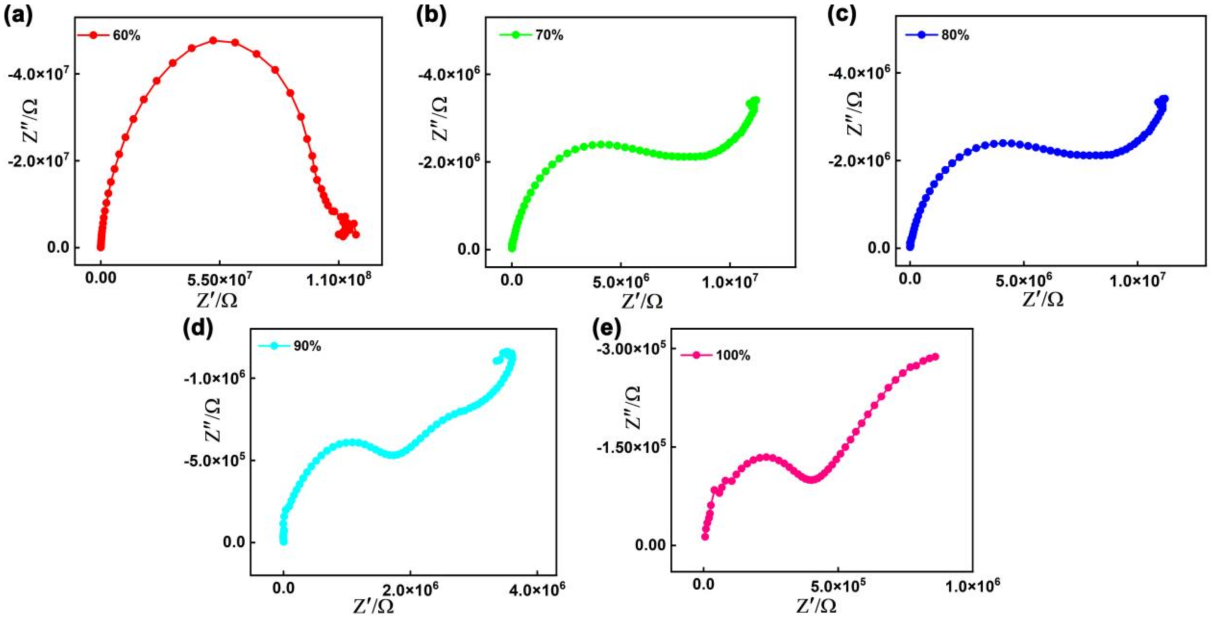

Fig. S8. Nyquist plot for **1** at 35 °C under (a) 60%, (b) 70%, (c) 80%, (d) 90%, (e) 100% RH.

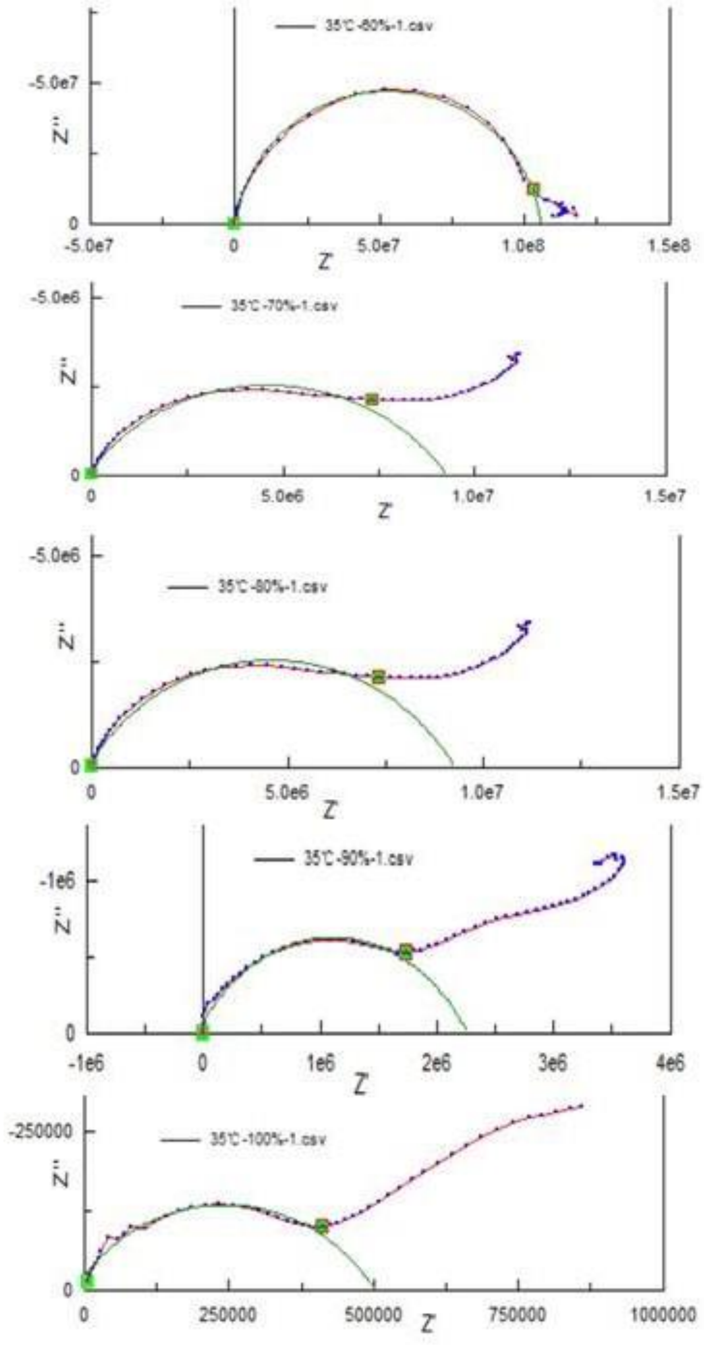

Fig. S9. The best-fit result of Nyquist plot for **1** at 35 °C under different RH levels.

Table S5. The proton conductivity of **1** at 35 °C under variable relative humidity (RH).

RH / %	R / S	σ / S cm^{-1}
60	1.06×10^{8}	1.79×10^{-8}
70	5.18×10^{7}	3.66×10^{-8}
80	9.42×10^{6}	2.01×10^{-7}
90	2.32×10^{6}	8.18×10^{-7}
100	5.23×10^{5}	3.63×10^{-6}

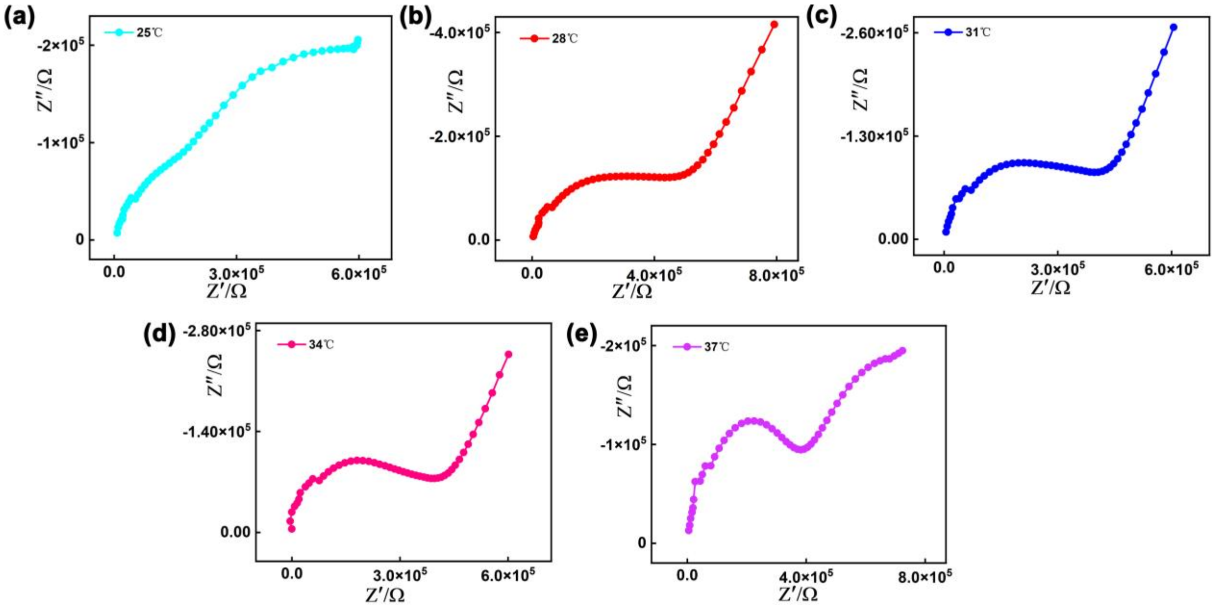

Fig. S10. Nyquist plot for **1** at (a)25 °C, (b) 28 °C, (c) 31 °C, (d) 34 °C and (e) 37 °C under 100% RH.

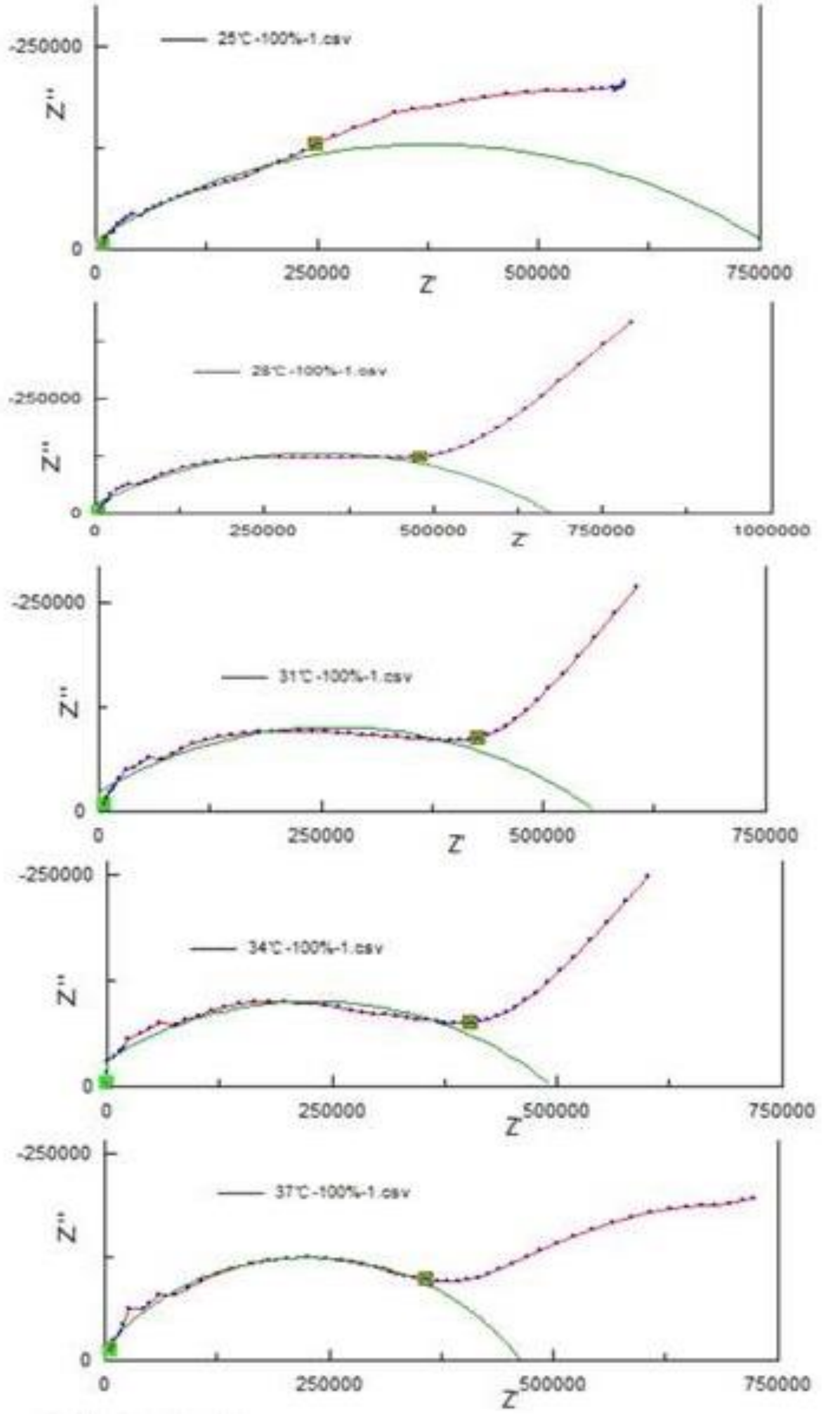

Fig. S11. The best-fit result of Nyquist plot for **1** at different temperatures under 100% RH.

Table S6. The proton conductivity of **1** at 100% RH under variable temperature.

T / ℃	*R* / *S*	σ / S cm^{-1}
25	7.52×10^{5}	2.52×10^{-6}
28	6.80×10^{5}	2.79×10^{-6}
31	6.14×10^{5}	3.09×10^{-6}
34	5.37×10^{5}	3.53×10^{-6}
37	4.75×10^{5}	4.00×10^{-6}

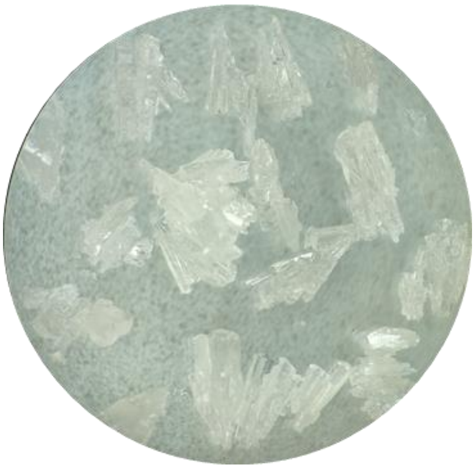

Fig. S12. The photograph of crystals of complex **1** after exposed to 25−37 ^{o}C and 60−100% RH conditions during the whole proton conductivity measurements.

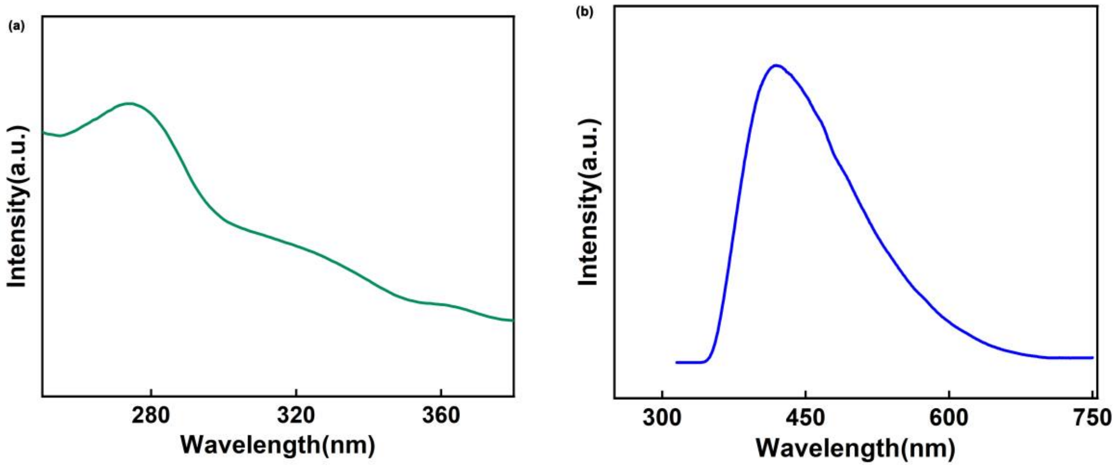

Fig. S13. Solid-state excited (a) and emission (b) spectra free Htza ligand.

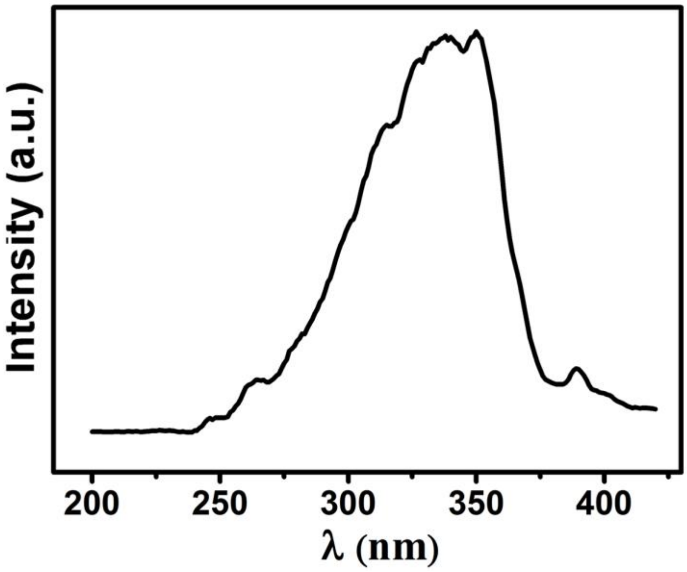

Fig. S14. Solid-state excitation spectra for **1** at room temperature.

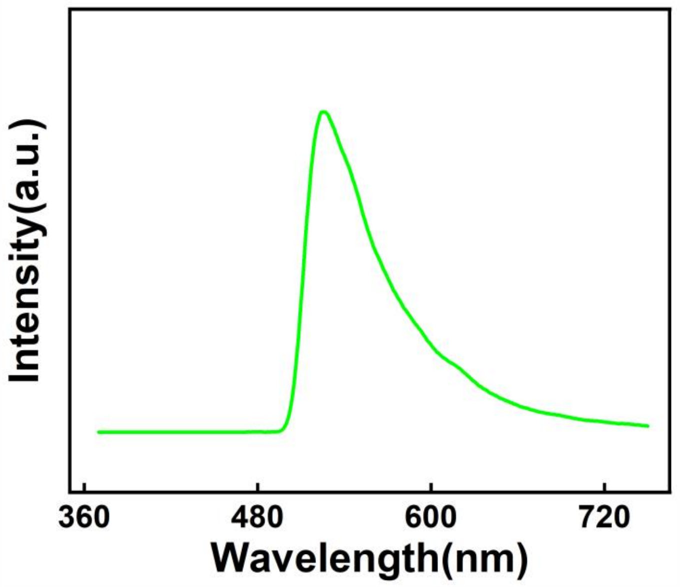

Fig. S15. Soild-state emission spectra exicited upon 350 nm of the free 1-Htza ligand at room-temperature.

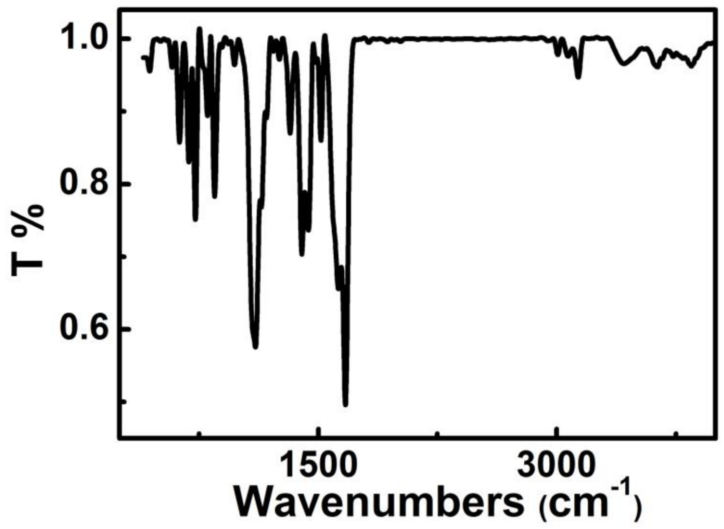

Fig. S16. IR spectra for **1**.
